# Metabolic Alterations of Thyroid Cancer as Potential Therapeutic Targets

**DOI:** 10.1155/2017/2545031

**Published:** 2017-11-06

**Authors:** Domenico Ciavardelli, Maria Bellomo, Ada Consalvo, Caterina Crescimanno, Veronica Vella

**Affiliations:** ^1^School of Human and Social Science, University “Kore” of Enna, Enna, Italy; ^2^Centro Scienze dell'Invecchiamento e Medicina Traslazionale (CeSI-Met), Chieti, Italy; ^3^Endocrinology Section, Department of Clinical and Experimental Medicine, Garibaldi-Nesima Hospital, University of Catania, Catania, Italy

## Abstract

Thyroid cancer (TC) is the most frequent endocrine tumor with a growing incidence worldwide. Besides the improvement of diagnosis, TC increasing incidence is probably due to environmental factors and lifestyle modifications. The actual diagnostic criteria for TC classification are based on fine needle biopsy (FNAB) and histological examination following thyroidectomy. Since in some cases it is not possible to make a proper diagnosis, classical approach needs to be supported by additional biomarkers. Recently, new emphasis has been given to the altered cellular metabolism of proliferating cancer cells which require high amount of glucose for energy production and macromolecules biosynthesis. Also TC displays alteration of energy metabolism orchestrated by oncogenes activation and tumor suppressors inactivation leading to abnormal proliferation. Furthermore, TC shows significant metabolic heterogeneity within the tumor microenvironment and metabolic coupling between cancer and stromal cells. In this review we focus on the current knowledge of metabolic alterations of TC and speculate that targeting TC metabolism may improve current therapeutic protocols for poorly differentiated TC. Future studies will further deepen the actual understandings of the metabolic phenotype of TC cells and will give the chance to provide novel prognostic biomarkers and therapeutic targets in tumors with a more aggressive behavior.

## 1. Introduction

Thyroid cancer (TC) is the most frequent endocrine tumor with an increasing incidence worldwide [1, 2] in the last decades. The improvement of diagnosis by ultrasound scan may partially explain this increase. However, the rise in TC incidence accounts mostly for differentiated subtypes and for tumors of all sizes, suggesting that overdiagnosis cannot be the only explanation and that new risk factors have to be considered. Exposure to ionizing radiation [3], iodine-deficiency [4], and family history for TC are known risk factors. Of note, it has recently been suggested that modifications of lifestyle and environment risk factors might explain the rise in TC incidence [5].

TC originates from epithelial cells and is classified in four major groups that are papillary (PTC), follicular (FTC), poorly differentiated (PDTC), and anaplastic (ATC). Among them, PTC accounts for almost 90% of all thyroid tumors. PTC frequently metastasize to local lymph node metastasis [6] while FTC often metastasize to distant organs such as lung and bone. Nevertheless, differentiated cancers (PTC and FTC) usually grow slowly and have good prognosis. Indeed survival rate after 5 years is 98% for PTC [7]. In contrast, ATC has a median survival rate of 3–5 months [8]. Medullary TC (MTC) originates from parafollicular C cells [9], has neuroendocrine features, and has a frequency that is about 3–5% of all thyroid tumors. Usually patients with MTC have a good 10-year survival rate that reaches 75% [9].

Many studies have shown that thyroid tumors display different clinical behavior, even among the same pathological group. Indeed, in some cases, well differentiated carcinomas are more aggressive than expected, making it difficult to predict tumor clinical behavior. Ultrasound guided fine needle aspiration biopsy (FNAB), followed by histological examination, represents the first step based on which patient treatment and follow-up have to be defined. However, sometimes they are not sufficient to properly classify thyroid lesions and identify tumors with worst prognosis. Currently, FNAB has some limitations specifically concerning follicular lesions (labeled as “indeterminate” or “nondiagnostic”) and lesions suspected of malignancy according to Bethesda Thyroid Cytology Classification [10]. As a consequence there is an increasing need to add new methods in order to improve sensitivity and sensibility to TC diagnosis.

Treatment of TC includes thyroidectomy, radioactive iodine (at least until TC cells are still differentiated), and TSH suppression through thyroid hormone therapy. Surgery is the first step of TC therapy, and patients that have an intrathyroid disease can be treated only with thyroidectomy. Radioactive iodine is another treatment opportunity for differentiated TC. However, the required prerequisite is that cancer cells need to express the sodium-iodide symporter (NIS). Otherwise, the cytotoxic effect of radioactive iodine cannot be exerted. Besides, an important mainstay in the treatment of TC is TSH suppression via thyroid hormone therapy. Since TSH is an important growth factor for thyroid cells, its reduction by thyroid hormones is critical to slow cancer cell proliferation and prevent local and distant recurrences. All these treatments are not successful with MTC and ATC, as they are not responsive to TSH suppression or radioactive iodine.

During thyroid tumorigenesis, alterations of oncogenes and tumor suppressors lead to abnormal proliferation, resistance to apoptosis, enhanced angiogenesis, and increased invasiveness. As previously stated, highly aggressive and poorly differentiated TC are often characterized by impaired iodine uptake when they become refractory to radioiodine treatment, the standard therapeutic approach for most of the histological TC subtypes. Distant metastasis, frequently localized in lung and bone, is the major cause of mortality and occurs in 10% of patients.

Recently, much emphasis was given to the cancer stem cell (CSC) model to explain the heterogeneity within TC [11]. According to this model, there is a subpopulation of cancer cells, called CSCs, in TC [12, 13] with a self-renewal and multilineage potential. The CSC model can also explain recurrence, metastasis, and therapy-resistance as these cells are resistant to the conventional therapies. Moreover, cancer growth, stemness, and metabolic phenotype often share similar signaling pathways [14, 15].

Metastatic cells are often associated with the lack of radioactive iodine uptake ability since thyroid tumors undergo a dedifferentiation program [16]. However, specific inhibition of oncogenic pathways associated with TC progression is not always able to redifferentiate cancer cells in advanced TC. Therefore, the development of new therapeutic strategies is needed.

During the progression toward thyroid malignancy, significant metabolic alterations have been recently described in addition to histologic, cytological, and molecular abnormalities. Metabolomic studies performed on FNAB or surgical specimens showed significant differences in energy metabolism between normal and neoplastic thyroid tissues, as well as among different thyroid lesions. Hopefully, metabolomic profile of TC, in addition to the actual diagnostic criteria, may provide more mechanistic insight on TC onset and progression, thus indicating new therapeutic routes that finally help the clinical management of patients with TC.

## 2. Thyroid Cancer Metabolism: Warburg and “Reverse Warburg” Effects

Thyroid hormone synthesis is an oxidative highly requiring energy process. Mitochondria produce the majority of the energy necessary to maintain cell functions through the oxidative phosphorylation (OxPhos) that takes place within the inner mitochondrial membrane [17]. Under physiological conditions, OxPhos leads to a concomitant production of high levels of reactive oxygen species (ROS). For this reason, several endogenous antioxidant systems are found in the thyroid gland. In thyroid tumors, there is an increased oxidative damage due to defects in antioxidant systems [18]. Specifically, increased activity of the enzymes involved in the antioxidant defense has been observed in differentiated TC, while their inactivation is typical of poorly differentiated tumors [19].

Furthermore, it has been proposed that high proliferating cancer cells often show suppression of mitochondrial OxPhos and a shift toward a high rate of glycolysis, with increased glucose consumption and production of lactate despite adequate oxygen. This metabolic shift facilitates the growth and survival of cancer cells, under hypoxic and nutrient-depleted condition, by enhancing biosynthetic fluxes and antioxidant defense during rapid proliferation, and is known as “Warburg effect” [20]. This metabolic reprogramming is regulated by transcription factors such as the hypoxia inducible factor 1 alpha (HIF-1*α*) that activates the glycolysis and inhibits OxPhos [21, 22]. HIF-1*α* is not expressed in normal thyroid tissues but is overexpressed in aggressive types of TC such as ATC [23]. Overexpression of HIF-1*α* has been also associated with distant metastasis in PTC [24, 25]. Furthermore, HIF-1*α* activates the expression of glycolytic enzymes such as the hexokinase II (HKII), phosphoglycerate kinase (PGK), glucose-6-phosphate dehydrogenase (G6PDH), and lactate dehydrogenase A (LDH-A), or glucose and lactate transporters, such as glucose transporter 1 (GLUT1) and the monocarboxylate transporter 4 (MCT4), that have been found overexpressed in TC [26–29]. Overall, these findings suggest that some cells within thyroid tumors rely on aerobic glycolysis for energy production, thus exhibiting the Warburg phenotype.

**Figure 1:**
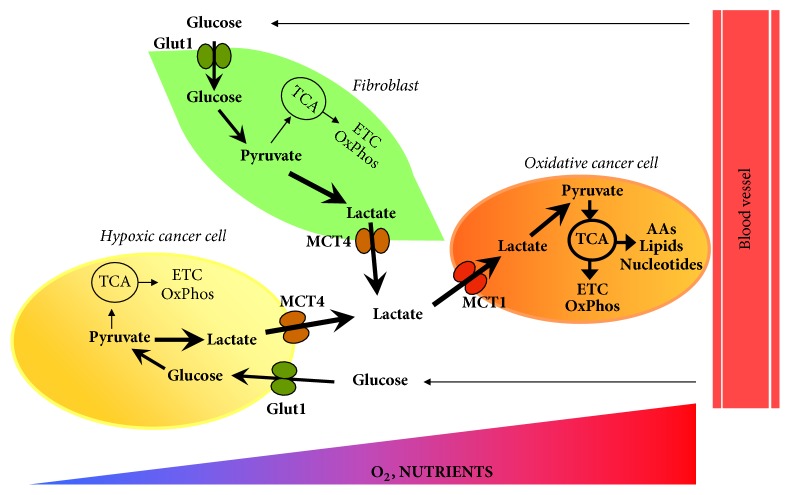
*Tumor microenvironment and multicompartment metabolism in thyroid cancer*. Oxidative cancer cells (shown in orange), growing under oxygen and nutrient-replete conditions, take up the lactate produced by cancer associated fibroblasts (CAFs, shown in light green) that exhibit a glycolytic phenotype. Cancer cells import lactate through the high affinity monocarboxylate transporter 1 (MCT1) while lactate export involves the low-affinity monocarboxylate transporter 4 (MCT4) in CAFs. Oxidative cancer cells convert lactate in pyruvate that is used for mitochondrial energy production. Experimental evidences support this metabolic symbiosis between cancer cells and CAFs in TC. Furthermore, in several cancer types, cancer cells growing under hypoxic and nutrient starved conditions (shown in light yellow) are more glycolytic compared with oxidative cancer cells and provide them with lactate to fuel tricarboxylic acid cycle and mitochondrial adenosine 5′-triphosphate production. This model needs to be evaluated in TC. ETC: electron transport chain; Glut1: glucose transporter 1; MCT1: monocarboxylate transporter 1; MCT4: monocarboxylate transporter 4; OxPhos: oxidative phosphorylation; TCA: tricarboxylic acid cycle.

TC cells coexist with different type of host cells like fibroblasts, cells of the immune system, and endothelial cells, in the so-called tumor microenvironment. Accumulating evidences support the idea that stromal cells such as cancer associated fibroblasts (CAFs) support the metabolic reprogramming of cancer cells. It has been proposed that cancer cells activate glycolysis and expression of the low-affinity MCT4 in neighboring CAFs, which provide high-energy metabolites such as lactate to support the metabolic requirement of proliferating cancer cells. Lactate, exported via MCT4 by CAFs, is taken up by cancer cells through the high affinity monocarboxylate transporter 1 (MCT1) and converted in pyruvate that is used for mitochondrial oxidative metabolism. This coupling of stromal catabolism and anabolic growth of cancer cells is called “reverse Warburg effect” [30]. Recently, the expression profile of MCT4, MCT1, and translocase of outer mitochondrial membrane 20 (TOMM20), a marker of mitochondrial mass, has been investigated in PTC and ATC [31, 32]. High expression levels of both MCT1 and TOMM20 have been found in ATC while TOMM20 has been found upregulated in PTC compared with noncancerous thyroid tissue. Furthermore, MCT4 has been found to be overexpressed in stromal cells associated with advanced PCTs and ATC [27]. These findings indicate that metabolic coupling between stromal and cancer cell occurs in TC microenvironment (Figure 1).

## 3. Oncogene Activation and Thyroid Cancer Metabolism

Different studies clearly showed that cancer metabolic reprogramming, controlled by oncogenes and other tumor-related molecules, is a critical factor determining the clinical phenotypes of TC. Furthermore, metabolic alterations linked to oncogene mutations (see Table 1) are able to affect the aggressiveness of TC.

The mutation BRAF^V600E^ of the gene encoding for the serine/threonine-protein kinase B-raf is the most frequent activating mutation in PTC occurring in approximately 45% of PTC [40]. The T1799A transverse point mutation of BRAF gives rise to a constitutive activation of the serine/threonine kinase of the corresponding protein. Previous studies have demonstrated that BRAF^V600E^ is expressed by PTC with aggressive pathological features, increased recurrence, loss of radioiodine avidity, and treatment failures [41]. Interestingly, the mutated BRAF^V600E^ is associated with a Warburg phenotype in PTC. Feng et al. found that the expression of the isoform M2 of pyruvate kinase (PKM2) correlates with the presence of BRAF^V600E^ and the aggressive tumor features of PTC. They observed that PKM2 overexpression confers a selective growth advantage to TC through activation of glycolysis [33]. In addition, Lee et al. reported a mitochondrial localization of BRAF^V600E^ associated with antiapoptotic effects and metabolic modifications. In accordance with previous studies, they reported that glucose uptake increases while O_2_ consumption decreased, thus suggesting the inhibition of OxPhos [35]. Recently, Nahm et al. [27] demonstrated that TC subtypes differently express proteins involved in glucose transport and catabolism. Analyzing a group of 566 thyroid carcinoma samples they found that the expression levels of GLUT1, MCT4, and HKII were associated with cancer aggressiveness and poor prognosis. Indeed, PTC samples harboring BRAF^V600E^ mutation and ATC showed the highest expression levels for these proteins. Aggressive tumors, as suggested above, probably express the highest GLUT1 and MCT4 levels because of their increased need for glucose to fuel proliferation. Glutamine metabolism alteration has been also found in cancers [34, 42]. By microarray and immunohistochemical staining, Kim et al. [34] observed a different glutamine metabolism-related protein expression in TC subtypes. They reported that glutamine metabolism alteration involves stromal cell dysfunction. Both ATC and “BRAF^V600E^ positive” PTC showed the high expression rate of proteins involved in glutamine metabolism.

The Rat Sarcoma (RAS) gene is the second most mutated gene in TC. RAS mutation causes the loss of its GTPase activity thus maintaining RAS in a constitutive active GTP-bound condition. Normally RAS is able to activate both mitogen-activated protein kinase (MAPK) and phosphatidylinositol 3-kinase/Akt (PI3K-Akt) pathways. However, RAS mutations constitutively activate the PI3K-Akt pathway in TC [36, 43]. Akt, stimulated by PI3K, rapidly accumulates in mitochondria where it phosphorylates and inhibits the *β*-subunit of adenosine 5′-triphosphate (ATP) synthase, thus suppressing OxPhos. Moreover, RAS mutations contribute to the initiation of undifferentiated thyroid cancer through MYC upregulation and the consequential Pax8 repression [44].

Phosphatase and tensin homologue deleted on chromosome ten (PTEN) and tumor protein 53 (TP53) are tumor suppressors [45–47] frequently deleted or mutated in TC [48–50]. Both of them are key regulators of glucose metabolism, as they modulate cell glucose uptake by regulating the membrane translocation of GLUT1 [51]. It has been also shown that PTEN and TP53 regulate the expression of several glycolytic enzymes, inhibiting the fermentative glycolysis and promoting glucose mitochondrial oxidation [52, 53]. Moreover, it is known that, downregulating PI3K/AKT pathway, PTEN reduces cell proliferation rate [45] while TP53 activation causes cell cycle arrest and apoptosis [47]. As a consequence, abrogation of either PTEN or TP53 functions increases both glucose cell uptake and glycolysis, making cancer cells more resistant to the metabolic stress caused by hypoxia and glucose deprivation [51]. So far cancer cells increase cell migration, proliferation, and resistance to apoptosis. Since enhanced glucose uptake is typical of poorly differentiated TC that have already loosened the radioiodine uptake ability, we can also state that glucose uptake is inversely correlated with iodine uptake ability [28, 54, 55]. Increased glucose uptake makes TC cells detectable through ^18^F-fluorodeoxyglucose positron emission tomography.

Tyrosine kinase receptors (RTKs) are frequently upregulated, or abnormally activated, in both PTC and ATC [43], even in the absence of ligands [56, 57]. Kachel et al. [58] demonstrated that, in TC, glycolytic enzymes such as PKM2 and LDH-A can be phosphorylated in a RTK manner, thus contributing to increasing proliferation under hypoxia condition and promoting the Warburg effect. Moreover, RTKs interact with each other [59, 60] and mediate the signaling from the extracellular matrix [61] thus controlling cancer cell behavior in terms of cell proliferation, survival, migration, and invasion.

## 4. The Current Status of Metabolomics in TC Research

Metabolome consists of the entire set of small molecules metabolic products in a biological system such as cells, tissues, organ, and biological fluids. Its composition may change in response to enzyme levels and activities, cellular regulation, signaling pathway activation, and genetic variations. Metabolomics has gained great importance in cancer research. In fact, as activation of specific metabolic pathways, such as aerobic glycolysis and glutaminolysis, has been found in many oncological diseases; cancer is now increasingly viewed as a metabolic disorder [62, 63]. Of note, alterations of specific small molecules called “oncometabolites” have been found in many tumors and are significantly involved in the development of malignancy [64]. Genomics, transcriptomics, and proteomics have strongly contributed to the knowledge of TC metabolism. However, this issue is beyond the scope of this review. One limitation of genomics and proteomics is that they do not completely characterize the actual cancer phenotype that, in contrast, is most closely connected to the cancer metabolome [65]. From this point of view, metabolomics can help, not only to discriminate different types of thyroid lesions, but also to provide fundamental mechanistic insight into thyroid carcinogenesis.

Using analytical techniques such as proton magnetic resonance spectroscopy (NMR) or mass spectrometry and multivariate statistical approaches, several studies showed higher level of intermediates of glucose, amino acid, and lipid metabolism in thyroid malignancy compared with healthy adjacent thyroid tissues.

Compared with healthy tissues or benign thyroid nodules, malignant lesions are characterized by increased levels of lactic acid, often paralleled by alanine, regardless of TC subtypes [66–71]. The high production of lactic acid is a common feature of many types of tumors exhibiting the Warburg phenotype. Currently, it is widely accepted that lactate is far from being a simple metabolic waste product and has several roles in cancer biology [72]. As discussed above, shuttling of lactate between stromal nontumor cells and cancer cells is a relevant metabolic feature of tumor microenvironment in TC. Furthermore, it has been recently shown that lactate is an important source of carbon for lipid biosynthesis during proliferation of fermentative cancer cells [73]. Lactate is also a potent signaling molecule involved in angiogenesis [74, 75]. Interestingly, when compared with healthy tissues, TC shows increased expression levels and activation of LDH-A, the key enzyme of lactate production in cells [26, 58].

Alteration of several amino acids other than alanine has been found in TC. Glutamine levels increase in malignant lesion compared with healthy adjacent tissue [69]. Tumor cells frequently show increased uptake of glutamine that provides nitrogen for protein and nucleotide synthesis, activate the regulator of protein translation mammalian target of rapamycin (mTOR), and provide intermediates of the tricarboxylic acid (TCA) cycle through anaplerosis for biosynthetic pathways [76]. Furthermore, it has been recently demonstrated that, in colon cancer cells, the high glycolytic rate associated with the Warburg phenotype and the generation of alanine from the glycolytic end product pyruvate activate anaplerosis and facilitate the entry of glutamine carbon into the TCA cycle to provide biosynthetic molecules [42]. This mechanism may explain the correlation found between alanine and glutamine levels in TC. Of note, increased expression rate of glutamine metabolism-related-proteins has been recently reported in TC [34].

High levels of serine and glycine were found in both FNA specimens from malignant nodules [71] and cancer thyroid tissues [67, 69] compared with control samples. Serine and glycine are interconnected in the so-called “one carbon metabolism” that generates diverse intermediates of lipid, nucleotide, and protein biosynthesis and is involved in cancer pathogenesis [77, 78]. Of note, the glycolysis intermediate 3-phosphoglycerate can be shunted from glycolysis into de novo biosynthesis of serine and thus of glycine, a process that is activated in highly glycolytic cancer cells. Furthermore, the expression rates of serine/glycine metabolism-related proteins have been recently found to be different among TC types and higher in BRAF^V600E^ variant of PTC [79].

Several studies also report significant alteration of lipid profiles in TC compared with adjacent nontumor tissues or benign lesions. Indeed, malignant TC show higher levels of saturated fatty acids such as myristic and palmitic acids [68], monounsaturated fatty acids such as palmitoleic and oleic acids, and specific monounsaturated phosphatidylcholines such as 1-palmitoyl-2-oleoylphosphatidylcholine (PC34:1) and 1-myristoyl-2-erucyl-sn-glycero-3-phosphocholines (PC36:1) [80, 81]. Higher levels of these unsaturated PCs have been recently found in several cancer tissues compared with adjacent healthy tissue [80, 82]. Increased choline levels have been reported in TC by NMR studies [71, 83, 84] and may further contribute to phospholipid biosynthesis. Interestingly, compared with healthy tissues, PTC and ATC show also increased expression levels of key enzymes involved in FA biosynthesis such as the fatty acid synthase and stearoyl-coenzyme A desaturase 1, which catalyzes the conversion of saturated fatty acids in monounsaturated fatty acids [68, 81]. Furthermore, it has been shown that malignant thyroid lesions exhibit lower levels of citrate compared with benign nodules [68, 71]. Overall these findings suggest that TC rely on de novo fatty acid biosynthesis and show high desaturase activity. In addition, lipid composition of TC subtypes (PTC, FTC, MTC, and ATC) was able to discriminate between them suggesting potential diagnostic application of lipid profiling [70].

**Figure 2:**
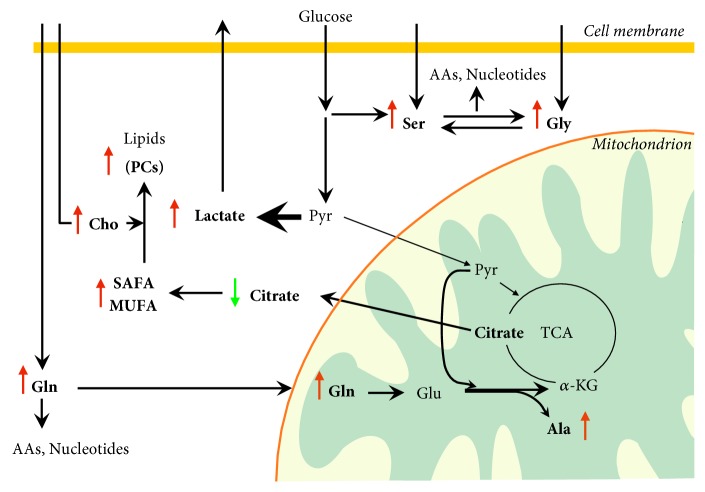
*Metabolomic alterations and active metabolic pathways in proliferating thyroid cancer cells*. Metabolomic analysis of thyroid cancer (TC) tissues indicates increased glycolytic rate, lactate production, glutaminolysis, and lipid biosynthesis. TC cells upregulate glucose uptake through the overexpression of the glucose transporter 1 (GLUT1). The pyruvate generated by the glycolysis is diverted toward lactate production through the action of lactate dehydrogenase A (LDH-A), overexpressed in TC and kept away from mitochondrial oxidative metabolism. Lactate is exported from tumor cells via a monocarboxylate transporter. The extracellular lactate activates the angiogenesis and provides metabolic fuel to proliferating cancer cells. The increase of serine and glycine may be explained by either increased uptake via neutral amino acid transporters or de novo biosynthesis of serine from the glycolytic intermediate 3-phosphoglycerate. Serine can be converted to glycine by the action of serine hydroxymethyltransferase that shows higher expression rate in TC. Therefore, de novo serine biosynthesis sustains glycine biosynthesis. The so-called “one carbon metabolism,” including both serine and glycine, cycles carbon units from amino acids to support amino acid and purine biosynthesis. Glutamine, also involved in amino acid and nucleotide biosynthesis, is taken up into the cell through the glutamine importer ASCT2 and is deaminated in mitochondria by glutaminase to form glutamate. Glutamate dehydrogenation produces alpha-ketoglutarate to replenish the tricarboxylic acid cycle intermediates. The enzymes involved in glutaminolysis have been found overexpressed in TC. It is still unclear whether the glutamate transamination with pyruvate to alanine and alpha-ketoglutarate, recently demonstrated in colon cancer cells, occurs in TC. Conversion of alpha-ketoglutarate to citrate supports the biosynthesis of cytosolic acetyl-CoA and, then, of saturated and monounsaturated fatty acids. Accordingly, increased expression levels of fatty acid synthase and desaturases have been found in TC. The increase of choline levels and the activation of fatty acid biosynthesis fuel the biosynthesis of lipids such as phosphatidylcholines. Green and red arrows indicate lower and higher metabolite levels, respectively, in TC compared with nontumor lesions or adjacent normal tissue. AAs: amino acids; Ala: alanine; *α*-KG: alpha-ketoglutarate; Cho: choline, Gln: glutamine; Glu: glutamate; Gly: glycine; MUFA: monounsaturated fatty acids; PCs: phosphatidylcholines; Pyr: pyruvate; SAFA: saturated fatty acids; Ser: serine; TCA: tricarboxylic acid cycle.

Finally, metabolomics suggests that TC is associated with high glycolytic rate and activation of biosynthetic pathways, compared with normal thyroid tissues (Figure 2). This phenotype is common to cancer cells and has been widely investigated, providing potential therapeutic targets. However, it is still unclear whether these metabolic features are mainly associated with malignant thyroid cells or tumor associated stromal cells. Furthermore, only little data are currently available on the metabolic differences between well differentiated and poorly differentiated TC subtypes. Consequently, these topics deserve further investigation to tentatively identify new metabolic targets and develop additional strategy aimed to synergistically improve the efficacy of standard therapy for iodine refractory TC.

## 5. Targeting Thyroid Cancer Metabolism: Challenges and Perspectives

In the last decades, the study of cancer metabolism has gained great attention, aiming to identify new potential therapeutic targets and early diagnostic or prognostic markers of oncological malignancies. Several preclinical or clinical studies are currently ongoing to determine the efficacy of inhibition of enzymes or transporters involved in the metabolic reprogramming of cancer cells [85, 86].

Quite surprisingly, therapeutic interventions targeting the TC metabolism have been investigated only in few studies. As discussed above, TC and stromal cells coexist in the tumor microenvironment interacting with each other, while maintaining their own metabolic phenotype. This metabolic heterogeneity suggests that more than one metabolic pathway should be targeted at the same time in order to improve antioncogenic effects of chemotherapeutics in TC. Therefore, it is intriguing to speculate that the inhibition of both aerobic glycolysis in thyroid CAFs and mitochondrial activity of TC cells could synergistically inhibit the growth of TC. Indeed, it has been hypothesized that the combination of the inhibition of lactate uptake and/or export through MCT1 and MCT4, respectively, with the suppression of mitochondrial activity may significantly affect the intimate interaction between TC cells and microenvironment resulting in improved antitumor activity [87]. Of note, the blockade of lactate import or export in cancer has been evaluated in preclinical study and inhibitors of MCT1 have entered clinical development. However, this hypothesis will need to be verified in future research.

Interestingly, it has been recently shown that inhibition of aerobic glycolysis using the hexokinase inhibitor 2-deoxyglucose improves the antiproliferative effect of metformin, a well-known inhibitor of mitochondrial electron transport chain, in TC cell lines. This finding indicates that the inhibition of glycolysis may be effective against TC cells itself regardless of tumor microenvironment [88].

Furthermore, several recent evidences show that the metabolic heterogeneity of solid tumors is associated with the metabolic difference not only between the tumor stroma and cancer cells but also between cancer cells that grow under nutrient and oxygen-replete or hypoxic and nutrient starved conditions [89, 90]. Tumor cells located close to blood vessels are thought to be mainly oxidative and use oxygen, glucose, and other energy substrates to produce ATP via OxPhos to feed anabolic pathways. In contrast, tumor cells that grow under hypoxic conditions are thought to be mainly glycolytic and release lactate. Lactate is taken up by oxidative cancer cells, converted in pyruvate by the isoform B of lactate dehydrogenase (LDHB), and used for mitochondrial ATP production [91, 92] (Figure 2). This metabolic symbiosis has been found in several cancers but needs to be still evaluated in TC.

However, it is worthy of note that, accordingly with metabolomic data, many glycolytic enzymes have been found to be overexpressed in advanced and poorly differentiated TC. For example, the overexpression PKM2 has been associated with tumor aggressiveness and negative prognosis of PTC [33]. Furthermore, undifferentiated TC show higher phosphorylation degrees of PKM2 compared with differentiated TC. PKM2 phosphorylation is associated with the Warburg phenotype and increased glycolytic flux [58]. Pyruvate kinase (PK) catalyzes the conversion of phosphoenolpyruvate to pyruvate, a rate limiting step of glycolysis. PKM2 is less active than PKM1 and is often overexpressed in cancer cells. The overexpression of PKM2 in cancer, associated with increased glycolytic rate and the Warburg phenotype, leads to the accumulation of glycolytic intermediates that, in turn, activate biosynthetic pathways supporting the cell proliferation [93]. Of note,* in vitro* and* in vivo* anticancer activity of PKM2 inhibitors has been assessed in preclinical studies and phase II clinical trials for various solid tumors [94]. Therefore, it is intriguing to speculate that the inhibition of PKM2 may contribute to overcoming the low efficacy of standard protocols in the therapy of iodine refractory TC.

Based on metabolomic and protein expression data, proteins involved in the lactate production (LDH-A), serine and one carbon metabolism (phosphoglycerate dehydrogenase and serine hydroxymethyltransferase 2), glutaminolysis (glutaminase), and fatty acid biosynthesis (fatty acid synthase), have been proposed for cancer therapy [85] and may also represent attractive therapeutic targets in TC.

Furthermore, it has been suggested that targeting CSCs metabolism may further improve the outcome of cancer therapy [95]. CSCs show distinct metabolic phenotype that can be either glycolytic or oxidative, depending on cancer types or metabolic adaptability to changes in microenvironment [96]. As discussed above, CSCs have been isolated in TC. However, metabolic alteration of thyroid CSCs still remains under investigation and will be of major interest to develop combined strategies aimed to suppress thyroid CSCs metabolism, their chemoresistance, and, thus, their metastatic ability.

Given the wealth of clinically available agents that target cancer metabolism, it is desirable that future studies would put more focus on the inhibition of energy metabolism as an alternative strategy for refractory TC therapy.

## 6. Conclusions

Recently, increasing studies have provided new insight into the metabolic adaptations of cancer cells. Indeed cancer has been recently defined as a “metabolic disease” since malignant cells generally show metabolic modifications in order to generate more energy to support their increased proliferation rate. To this purpose, more aggressive TC show increased glucose uptake, thus modifying their metabolism toward anabolism. Nevertheless, TC cells have multiple hyperactive metabolic pathways so that they are able to reprogram their metabolism upon nutrient deprivation conditions such as those occurring in hypoxic tumor microenvironment.

Within TC different metabolic compartments can be distinguished. In this view, cancer and stromal cells define two metabolic compartments in which stroma supports the metabolic requirement of TC cells promoting a tumor aggressive behavior. In addition, it is possible that the existence of heterogeneous populations of TC cells with different metabolic phenotype may be associated with the development of the resistance of TC to radioactive iodine therapy.

Thus, altering the balance between cancer and stromal cells or the metabolic cooperativity among different TC cell populations is a promising therapeutic strategy but still needs to be further studied. Moreover, these metabolic modifications have to be correlated to the specific thyroid molecular alterations in order to better elucidate the mechanisms of TC progression.

Finally, to give a therapeutic option to poorly differentiated and refractory TC, a better characterization of the metabolic phenotype of TC subtypes is clearly needed.

## Figures and Tables

**Table 1:** *Association between oncogene mutations and metabolic phenotype of thyroid cancer*. The table summarizes the association between oncogene activation or tumor suppressor inactivation and alterations of proteins involved in glucose uptake, glycolysis, and glutamine metabolism in thyroid cancer.

Thyroid oncogene	Target genes/metabolic pathways	Regulation	Reference
BRAF^V600E^	GLUT1, MCT4, HKII, CAIX, PKM2, GLS1, GDH, ASCT2	Activated	[27, 33, 34]
	O_2_ consumption	Inhibited	[35]

Ras mutations	ATP-synthase	Inhibited	[36]

PTEN loss of function	GLUT1	Activated	[37]

p53 mutations	GLUT1	Activated	[38, 39]

ASCT2: amino acid transporter-2; CAIX: carbonic anhydrase IX; GLS1: glutaminase; GLUT1: glucose transporter 1; GDH: glutamate dehydrogenase; PKM2: pyruvate kinase isoform M2.
